# Neoantigens in Chronic Obstructive Pulmonary Disease and Lung Cancer: A Point of View

**DOI:** 10.1002/prca.201800093

**Published:** 2019-02-11

**Authors:** Lona Zeneyedpour, Lennard J. M. Dekker, Jenny J. M. van Sten‐van`t Hoff, Peter C. Burgers, Nick H. T. ten Hacken, Theo M. Luider

**Affiliations:** ^1^ Department of Neurology Erasmus MC Rotterdam 3015 GE Netherlands; ^2^ Department of Pulmonology University Medical Center Groningen/University of Groningen 9713 Groningen Netherlands

**Keywords:** COPD, lung cancer, neoantigens

## Abstract

The goal of this manuscript is to explore the role of clinical proteomics for detecting mutations in chronic obstructive pulmonary disease (COPD) and lung cancer by mass spectrometry‐based technology. COPD and lung cancer caused by smoke inhalation are most likely linked by challenging the immune system via partly shared pathways. Genome‐wide association studies have identified several single nucleotide polymorphisms which predispose an increased susceptibility to COPD and lung cancer. In lung cancer, this leads to coding mutations in the affected tissues, development of neoantigens, and different functionality and abundance of proteins in specific pathways. If a similar reasoning can also be applied in COPD will be discussed. The technology of mass spectrometry has developed into an advanced technology for proteome research detecting mutated peptides or proteins and finding relevant molecular mechanisms that will enable predicting the response to immunotherapy in COPD and lung cancer patients.

## 1 Introduction

The importance of neoantigens for lung cancer (LC) is well acknowledged. However, it is interesting to research a possible role of neoantigens in chronic obstructive pulmonary disease (COPD) by using mass spectrometry (MS) technology. This manuscript starts with describing the role of neoantigens, followed by an introduction of COPD and lung cancer and an analysis of the link between COPD and LC.

Some data on mortality in COPD and LC give insight differences in developing and underdeveloped regions. A section on genomics is followed by an analysis of how to detect neoantigens by mass spectrometry.

## 2 Neoantigens

Neoantigens are antigens that deviate from own structures that change into coding protein structures that can be recognized by the immune system. They can be linked to deoxyribonucleic acid (DNA) repair mutations and generate increased tumor infiltrating lymphocytes (TILs). Neoantigens correlate with increased expression of multiple proinflammatory cytokines and immune‐related genes, MI‐polarized macrophage genes, programmed death ligand‐1 (PD‐L1) and programmed cell death‐1 (PD‐1).^1^ Identifying individual mutations by exome‐sequencing is desirable for developing neoantigen‐targeted cancer immunotherapies that aim to activate cytotoxic T cells and control tumor progression by major histocompatibility complex (MHC) molecules.^2^ T cells can recognize neoantigens and can mediate immune responses against tumor cells containing these neoantigens.^3^ To identify candidate neoantigens high‐throughput next generation sequencing (NGS) and whole‐exome sequencing (WES) is used.^4^ The use of WES combined with in silico peptide translation has become a promising approach to detect patient‐specific neoantigens.^5^

## 3 Chronic Obstructive Pulmonary Disease

COPD is a leading cause of death worldwide, smoke inhalation being widely accepted as one of the most important causes. COPD is characterized by chronic airflow obstruction in the lung and symptoms related to decreased expiratory volume.

An important modulator of the immune system is the regulatory T cell (Treg). Tregs are involved in the suppression of smoke induced specific immune response and a diminished presence or function of these cells may underlie the development of specific humoral immune response in COPD.^6^ Damage in the lung by COPD is caused by oxidative stress, inflammatory cytokine release, protease activity and auto‐antibody expression.^5^, ^7^ Shorter telomeres are associated with COPD and short telomere length may contribute to inflammation in COPD and increases susceptibility to emphysema.^5^ Systematic inflammation associated with COPD might cause an increase in apolipoprotein M (Apo M) expression and there are two single nucleotide polymorphisms (SNPs) flanking the Apo M gene involved that are associated with altered lung function.^8^ COPD is identified with an elevated reactive oxygen species (ROS) level and ROS are able to modify proteins by chemical side reactions (for instance by carbonylation), as well as signaling pathways and anti‐oxidant molecule functions. This can lead to activation in COPD of the mammalian target of rapamycin (mTOR) aging pathway via phosphoinositide 3‐kinase (P13K) activation by ROS, resulting in reduced antioxidant defense by FOXO3A inhibition and a loss of autophagy.^9^

Genome‐wide association studies (GWAS) have associated several SNPs which predispose an increased susceptibility to COPD and LC such as SERPIN2, HHIP, FAM13A, IREB2, CHRNA3, and CHRNA5.^10^

Single nucleotide variants in COPD observed in GWAS studies are for a part missense mutations, that generate differences in proteins that are hardly investigated on the protein level. The impracticality to measure large numbers of missense‐mutated peptides chosen from these GWAS studies hampers an assessment from a technical point of view. Also the possible presence of neoantigens in COPD and lung cancer^11^ is hampered by the sensitivity of mass spectrometry to detect and identify peptides derived of neoantigens and it is even debated if neoantigens exist in COPD.^12^

## 4 Lung Cancer

Lung cancer has become one of the leading causes of death with smoke inhalation as the main etiologic factor.^5^, ^13^ Lung cancer is caused by mutations in oncogenes,^5^ leading to the proliferation of mutated cells and the formation of a tumor. GWAS associated over 500 SNPs influencing cancer risk and downstream targets for at least three genes were enriched by cell cycle genes involved in G1/S transition. A history of emphysema is the highest risk factor for lung cancer among smokers.^14^ Molecular profiles in non‐small cell lung cancer generate ideas to develop molecular targeting agents that inhibit the growth signals resulting from driver mutations. Epidermal growth factor receptor (EGFR) and anaplastic lymphoma kinase inhibitors, such as PD1 immunotherapy, become the key drugs for lung cancer treatment.^2^ Immunological checkpoint blockade therapies targeting cytotoxic T‐lymphocyte antigen‐4 (CTLA‐4), PD‐1, and PD‐L1 have been shown to have remarkable benefits for the treatment of lung cancer.^2^ Research by Chae and co‐workers^1^ showed that the immunophenotype of lung adenocarcinoma (LUAD) can be seen as a primary infiltration by activated CD4 and CD8 cells.

## 5 The Link between COPD and Lung Cancer

COPD and lung cancer are interrelated diseases with substantial mortality and most probably an immunological link may exist between the two diseases.^15^ However, their pathophysiologic mechanisms are not yet fully understood.^16^ The increased risk of lung cancer in COPD patients suggests the existence of a two‐fold altered cell‐mediated immune response in COPD patients: dysregulation of T‐cells in the lungs and T‐cell exhaustion.^16^ Activation of nuclear transcription factor (NF)‐kB may have a crucial role in the development of lung cancer from COPD. NF‐kB activation increases the release of inflammatory mediators that can induce COPD, and also inhibits apoptosis, induces proliferation and accelerates cancer development.^14^The high prevalence of lung cancer in COPD suggests that there may be common mechanisms, such as premature aging in lung tissue, genetic predispositions to either disease or common pathogenic factors such as growth factors, activation of intracellular pathways or epigenetics.^5^ Various mechanisms to explain the association between COPD and lung cancer include genetic susceptibility, DNA damage and repair, epigenetics, downregulation of specific microRNA, expression of proinflammatory genes induced by hypoxia, tumor growth factor‐B and integrins, telomere length, and dysfunction.^17^ Telomere shortening is a risk factor in COPD and lung cancer.^5^

Chronic inflammation through the induction of several interleukins and cyclooxygenase‐2 activity may be an important player in the lung tumor formation among patients with COPD. For instance, CCL21 may favor cancer cell migration in the lungs of patients with COPD.^13^ Oxidative damage and antioxidant depletion may contribute to a greater risk to lung carcinogenis, especially in patients with underlying COPD.^13^ An epigenome wide association study identified that DNA methylation and repression of two genes—CCDC37 and MAP1B—was significantly associated with both COPD and lung cancer.^5^ COPD leads to changes in lipid profiles including increased ceramide levels in lung tissue mediated by high density lipoprotein.^8^ These changes in lipid metabolism in turn may alter other physiological responses, including the hypoxia response and EGFR signaling and may play a role in the link between COPD and lung cancer.^5^

STAT3 and its downstream genes, such as CBLN1, CBLN2, FGL1, FOX03, GJB1, HNF4α, TMEM27, and TTR, are differentially expressed in both LUAD and COPD.^18^ The tumor suppressor protein p53 is a general inhibitor of inflammation; its gene, TP53, is often mutated by cigarette smoke and may be suppressed by oxidant activation of NF‐kB mediated inflammation.^14^ Exposing human bronchial epithelial cells to high levels of particulate matter 2.5 (PM2.5; the mass per cubic meter of air of particles with a size of less than 2.5 μm) induces significant upregulation of vascular endothelial growth factor A (VEGA) production. Macroautophagy/autophagy is induced upon PM2.5 exposure and then mediates VEGA upregulation by activating the SRC‐CTA3 pathway in bronchial epithelial cells^19^

Mutational signature analysis in a study by Xiao and co‐workers^10^ suggests that there was no specific mutation pattern during the development of LC associated with COPD. The high concordance in the mutational burden further suggests that the inflammatory environment surrounding the tumor cells does not generate new mutations in LUAD patients, but integrative analysis of DNA methylation and transcriptome profiling demonstrates that the presence of COPD is associated with changes in methylation and expression in genes involved in immune response in non‐small cell lung carcinoma (NSCLC). Loss of encoding Parkin (PARK2) increases the expression of proinflammatory factors as well as nuclear NF‐kB, suggesting a role of PARK2 loss in inflammation, and PARK2 deficiency promotes genomic instability and cell transformation, so PARK 2 might have a suppressor role in the development of COPD and lung cancer.^20^

SNP variation‐associated inflammatory genes identified between COPD and lung cancer may play critical roles in a COPD‐LC transformation; activated nicotinic acetylcholine receptor gene in COPD may cause mutation and down‐regulates the expression of the crucial tumor suppressor gene TP53 and P53‐related signaling pathways, causing lung tumorigenesis.^21^, ^22^

Saber and co‐workers^23^ showed that COPD is not associated with the presence of KRAS mutations as observed in lung cancer, whereas presence of EGFR mutations was more frequent in non‐COPD as compared to COPD lung patients. Their findings that EGFR mutations are more common in non‐COPD lung patients might indicate that lung cancer development depends on activating EGFR mutations in non‐COPD patients. Lim and co‐workers^24^ argue that COPD is not a prognostic factor in advanced NSCLC patients, however, COPD had a negative impact on the overall survival of NSCLC patients in the smoker and stage IV subgroup.

GWAS have shown that large numbers of coding variants can have effects on the susceptibility of COPD.^25^ Although these coding variants have a significant effect on the susceptibility for COPD, the heterogeneity of the disease does not allow yet to translate this knowledge into clinical applicable molecular tools to identify those individuals which are susceptible for COPD and will develop lung cancer. Very recently, therapeutic antibodies that effect the immune system such as atezolizumab for lung cancer have been introduced with considerable results in the treatment of NSCLC compared to classical chemotherapy treatment (docetaxel, cisplatinum, gemcitabine) in case of advanced disease (for review see ref. 26). In general, for COPD no medication is available that cures the disease and the developed drugs for lung cancer have shown no effect in COPD although the number of studies on this topic is rather limited. It is suggested that an immunological link exists between the two diseases,^15^, ^16^ so detailed investigation can be highly rewarding for developing knowledge on possible treatment of COPD.

## 6 Mortality for COPD and LC

COPD and lung cancer are heterogeneous diseases that are for a large part (85% [man] and 69% [female] for instance in the Dutch situation [2018]) linked causatively by the use of cigarettes (Dutch national institute for public health and the environment; www.jellinek.nl).^16^, ^27^ In Table 1, the global situation and the situation in the continents is presented. From this table, one can notice that in the developed world (Europe, Oceania, and North America) similar ratios of mortality for COPD and lung cancer can be observed. In developing and underdeveloped regions the cause of COPD and lung cancer is much more linked to indoor pollution.^28^

**Table 1:** Annual mortality in COPD (2010) and lung cancer (2015)

Number of deaths^a^	COPD	Lung cancer	Ratio COPD/lung cancer
Global	2 837 877	1 823 929	1.55
Continents			
Europe	267 451	387 913	0.69
Oceania	10 256	11 822	0.87
North America	167 299	173 278	0.96
South America	117 865	62 922	1.87
Asia	2 159 952	1 068 862	2.02
Africa	103 325	37 748	2.71

a *Source*: For COPD: Burney et al. (2015).^29^ For lung cancer: WHO Globocan (http://www-dep.iarc.fr/WHOdb/WHOdb.htm).

## 7 Genomics

The recently published COPD Gene investigators study^30^ indicates that rarely found genetic variants were different in specific pathways such as the transforming growth factor beta pathway, the hedgehog pathway and the cilia‐related pathway in a relatively large cohort (*n* = 2543) of COPD patients and controls who were not affected by smoke. The criteria used for the patient group were GOLD grades 3 and 4 (https://goldcopd.org/; postbronchodilator forced expiratory volume [FEV] < 50% predicted), age less than 65 years, and for the control group frequency‐matched pack years of cigarette smoking, FEV1 > 80% predicted, age >65 years, no significant emphysema. For instance for proteins CTC1, OR5B12, GTF3C5, BLVRB, SLC7A7, SLC 26A7, and Notch2 coding mutations were associated with COPD.^30^ Most ideally patients can be categorized for these missense mutations and treated for COPD in a much earlier phase besides prevention and assistance in the cessation of smoke in a very early stage. In general, GWAS studies until now do not result into a molecular or a genetic clinical test because sensitivity and sensitivity is relative low.

The risk of developing lung cancer is eight times higher if COPD has been diagnosed.^27^, ^31^ Common molecular mechanisms related to inflammation, to innate immune responses and to carcinogenic processes are affected in COPD and lung cancer.^32^ These molecular mechanisms are most likely defense mechanisms to the chemical exposure of smoke in the lung. Research by Lambrechts and co‐workers showed that rs1051730 on chromosome 15q24/25 is associated with the presence and severity of emphysema and they discussed a shared pathogenic mechanism in COPD and lung cancer.^22^ As mentioned above, anti‐PD‐L1 antibody (e.g., atezolizumab) has revolutionized the treatment of NSCLC patients and has been approved in 2016^33^ by the U.S. Food and Drug Administration. For COPD such a treatment does not yet exist and therapeutic antibodies to proteins of the innate system (cytokines) have not proven to be successful.^34^ However, a better understanding of mechanisms of the development of COPD can hopefully lead to the finding of key regulated molecules that can be effectively targeted by drugs or therapeutic antibodies. Research by Mark and co‐workers^15^ showed that PD1 expression was increased in tumors of COPD patients and the presence of COPD was associated with longer progression‐free survival of patients treated with immune checkpoint inhibitors.

The enormous efforts in GWAS and cohort studies^8^, ^10^, ^20^ in which NGS is performed on cellular materials of patients with COPD and lung carcinoma open ways to investigate these pathways on a protein level,^35^ especially, if specific coding mutations or neoantigens specific for COPD or lung cancer can be identified.^30^ As a consequence the affected molecular mechanism (e.g., immune response or inflammation) can be targeted or modulated in a way beneficial for the patient.

## 8 Neoantigens and Mass Spectrometry of Missense Mutations

The presence of a high number of clonal neoantigens in homogeneous LUAD may favor immune surveillance, whereas in lung squamous cell carcinoma immune escape may be more prevalent through human lymphocyte antigen (HLA) downregulation. A high clonal neoantigen burden in LUAD is associated with an inflamed microenvironment with activated T cells, potentially regulated by inhibitory immune checkpoint molecules and their ligands.^36^ Immune checkpoint inhibitors have shown significant therapeutic responses against tumors containing increased mutation‐associated neoantigen load.^37^ The detection of these neoantigens is of interest. Direct proteomic analysis of MHC ligands by liquid chromatography and tandem mass spectrometry (LC‐MS/MS) enables discovery of these neoantigens directly from cancer cells.^38^ The success of checkpoint inhibitor therapies underlines the notion that tumor‐specific T cell responses pre‐exist in patients with lung cancer and are kept under tight control via immune modulatory mechanisms.^39^ In non‐small cell lung cancer, smoke‐related carcinogenesis is strongly associated with higher mutation rate and immunotherapy response, and the presence of neoantigen‐specific T cells in the peripheral blood demonstrates that some neoantigens are capable of inducing T cell reactivity.^3^

Recent proteomic approaches provide a comprehensive way to analyze whole HLA ligandomes containing various types of tumor‐associated antigens and direct peptide isolation from live cells using antibodies directed against HLA molecules followed by LC‐MS/MS sequencing is an ideal strategy to map and screen natural T‐cell epitopes presented by cancer cells.^40^

Neoantigen loss occurs through elimination of tumor subclones or through deletion of chromosomal regions containing truncal alterations and were associated with changes in T cell receptor clonality. There could be two mechanisms of neoantigen loss in a resistant tumor: 1) through the immune elimination of neoantigen‐containing tumor cells that represent a subset of the tumor population, and 2) through the occurrence of one or more genetic events in a tumor cell that results in neoantigen loss, followed by selection and expansion of the resistant clone.^37^ Frameshift mutation in neoantigens provide a unique opportunity to target common tumor‐suppressor genes such as TP53 and BAP1, and frameshift indels trigger an increased quantity of neoantigens and greater mutant binding specificity.^39^

Application of the epitope prediction approach to sequencing data from different cancer types reveals a range of predicted neoantigens per individual tumor, providing evidence that neoantigens are frequent in most human cancers.^41^

Mass spectrometry has, in addition to the potential to identify proteins in the presence or absence of databases, the inherently present possibility of quantification of proteins and peptides in a relatively sensitive way.^42^ This opens possibilities to detect missense mutations in antigens and even neo‐antigens in biopsies and body fluids.

By mass spectrometry coding mutations can be detected and quantified on the protein level and in heterozygous patients the ratio of the wild type and mutated protein can be determined.

**Figure 1:**
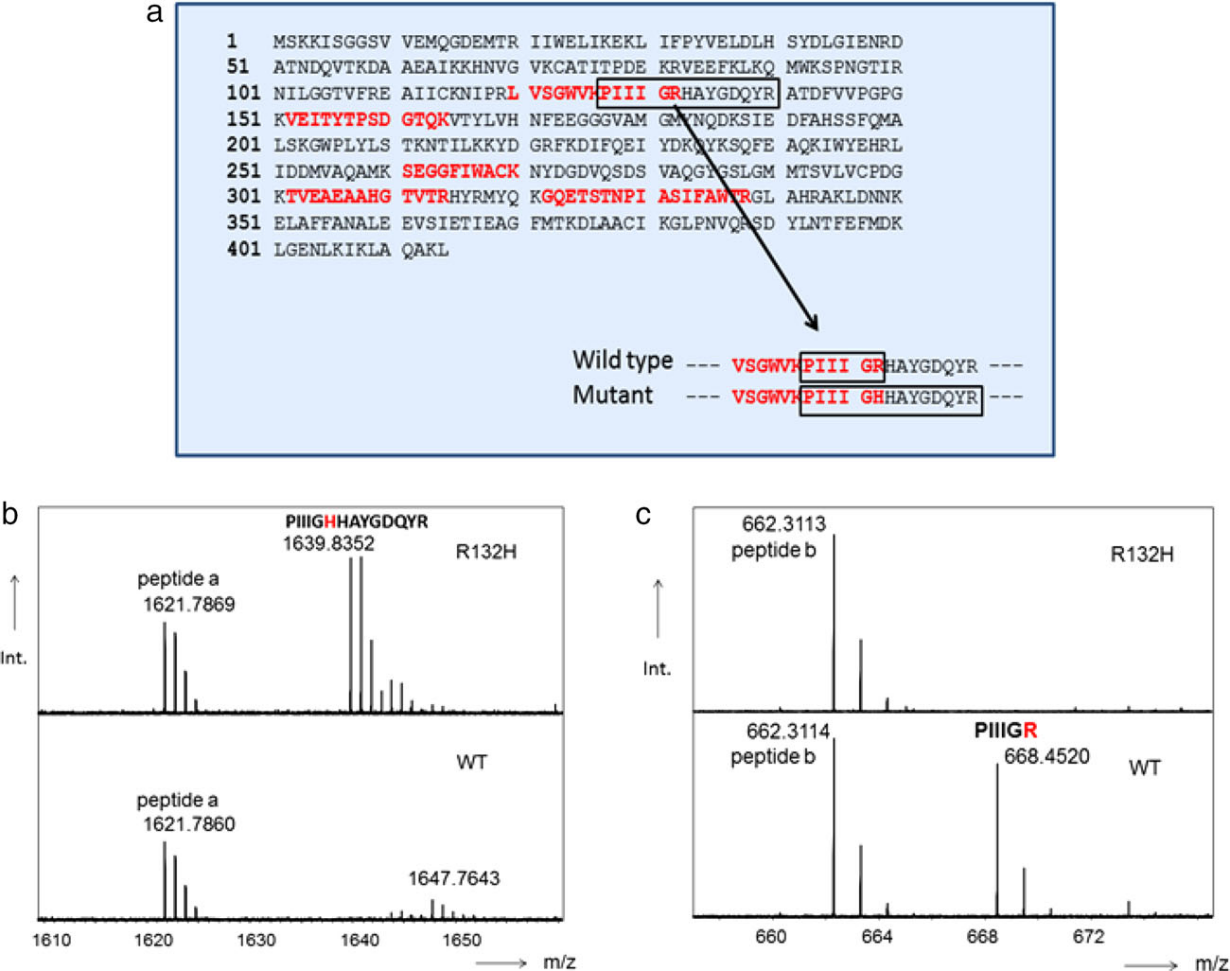
a) Primary structure of mutant IDH‐1. b,c) Partial mass spectra of mutated IDH‐1(top) and wild type (WT) IDH1 digested proteins. Partial sequence of mutant …RLVSGWVK | PIIIGHHAYGDQYR | ATDFVVPGPG… Partial sequence wild type …RLVSGWVK | PIIIGR|HAYGDQYR| ATDFVVPGPG… Trypsin cleavage sites are indicated with |. Peptides a and b are peptides from other proteins and they may serve as references for intensity variation observed between various samples.

It is particularly of interest if the abundance of these variants is influenced by the presence of COPD. Hereafter, an example is given of the detection of a mutation in isocitrate dehydrogenase (*IDH1*) on position 132 of this protein^43^ which is observed in a few specific tumors including lung carcinoma. The example in Figure 1 describes a mutation at positon 132, where an arginine (R) is replaced by a histidine (H). The difference in weight between the mutated and the normal peptide can be observed in the mass spectrum. However, the mutation dictates the size of the tryptic peptide and the composition of amino acids in the peptide. Sometimes specific enzymes (matalloendopeptidase [Lys‐N] or chymotrypsin) ought to be applied to technically realize the visualization of these mutated and normal peptides.^44^ Mass spectrometry allows to measure the ratio of mutated and the corresponding normal peptides potentially associated to a specific disease. These ratios can be determined accurately if synthetic stable isotope labeled peptides are applied.

Although these missense mutations can be attractive targets for therapy, neoantigens presented on human leucocyte antigens (HLA)^45^ might be even a more attractive way to find proteins that might be affected in COPD. Recently, Bassani‐Sternberg and co‐workers^46^ have shown the possibility to detect neo‐antigens in native human melanoma tissue. Neoantigens that are displayed by the MHC can be analyzed by mass spectrometry (MS) and it is of interest to determine the immunopeptidome in lung tissue of COPD patients. The feasibility of this approach as described by Bassani‐Sternberg and co‐workers^47^ shows that by MS, mutated peptide ligands for HLA can be identified in relative small periods of time (three weeks) in contrast to immunological oriented studies. Intensive fractionation of peptides eluted from HLA makes it possible to identify these neo‐epitopes in proteins by mass spectrometry in a much faster way and can lead potentially to targeted approaches or specific antibody treatment for COPD, indicating that MS technology has major advantages for detecting relevant proteins.

For absolute quantitative mass spectrometric analysis of mutated proteins and mutated peptides, a stable isotope labeled peptide or protein needs to be synthesized. In this way, a precise quantification can be reached for a mutated protein. Targeted mass spectrometry (selection reaction monitoring and parallel reaction monitoring) allows the measurement of tens to hundreds of mutated proteins in a single analysis. A disadvantage of this technique is the low sensitivity if no sample fractionation is performed for serum or tissue samples. Without sample preparation, most often microgram per mL biofluid can be reached. If affinity separations (e.g., by specific column chromatography materials, binders such as antibodies or affimers) are applied one can reach the ng mL^−1^ biofluid or pg protein per g tissue.^35^, ^42^ These improved sample preparations and improved technology in quadrupole and high resolution detection in advanced mass spectrometers (hardware and software) will enable the large scale detection and quantitation of mutated proteins in the near future.

Specific binders for cancer‐associated pathways can be very instrumental to extract differentially expressed proteins from these pathways in lung biopsies. Mass spectrometry has the advantage that it is possible to define very accurately the ratios of mutated proteins in these pathways and may show proteins that can effectively modulate immunological processes involved in the induction and progression of COPD.

## 9 Conclusion

The idea that cancer has pre‐stages is widely accepted (for reviews see refs. 5, 48). The same might hold for COPD and as the risk of lung cancer is eight times higher in COPD patients one may assume that in a part of the COPD patients COPD is a pre‐stage of lung cancer. If so, than neoantigens might also be present in COPD. Since the MS‐based technology has been described to identify these neoantigens, the possibility to investigate in depth these neoantigens is of high interest.

## Conflict of Interest

The authors declare no conflict of interest.
